# Management, Flow, and Outcomes of Patients with Aortic Stenosis Followed by a Heart Valve Clinic: The Untold “Behind the Scene” from a High-Volume, Real-World Experience

**DOI:** 10.3390/jcm14010267

**Published:** 2025-01-05

**Authors:** Federico Cammertoni, Natalia Pavone, Piergiorgio Bruno, Gabriele Di Giammarco, Francesco Burzotta, Enrico Romagnoli, Antonella Lombardo, Francesca Graziani, Marialisa Nesta, Maria Grandinetti, Serena D’Avino, Alberta Marcolini, Gessica Cutrone, Edoardo Maria D’Acierno, Rudy Panzera, Gabriele Mazzenga, Marco Montesano, Massimo Massetti

**Affiliations:** 1 Department of Cardiovascular Sciences, Fondazione Policlinico Universitario A. Gemelli IRCCS, 00168 Rome, Italy; natalia.pavone@policlinicogemelli.it (N.P.); piergiorgio.bruno@policlinicogemelli.it (P.B.); francesco.burzotta@policlinicogemelli.it (F.B.); enrico.romagnoli@policlinicogemelli.it (E.R.); antonella.lombardo@policlinicogemelli.it (A.L.); francesca.graziani@policlinicogemelli.it (F.G.); marialisa.nesta@policlinicogemelli.it (M.N.); maria.grandinetti@policlinicogemelli.it (M.G.); serenadavino93@gmail.com (S.D.); alberta.marcolini@guest.policlinicogemelli.it (A.M.); massettimas@yahoo.it (M.M.); 2 Faculty of Medicine and Surgery, Catholic University of the Sacred Heart, 00168 Rome, Italy; gabrieledigiammarco57@gmail.com (G.D.G.); ge.cutrone@gmail.com (G.C.); dacierno.edoardo@gmail.com (E.M.D.); panzerarudy@gmail.com (R.P.); gabrielemazzenga98@gmail.com (G.M.); marcomontesano3@gmail.com (M.M.); 3 Department of Neuroscience, Imaging and Clinical Science, School of Medicine and Health Science, Università “G. D’Annunzio” Chieti-Pescara, 66100 Chieti, Italy

**Keywords:** aortic stenosis, heart valve clinic, transcatheter aortic valve implantation, valvular heart disease

## Abstract

**Background:** According to current guidelines, patients with heart valve disease should be followed by Heart Valve Clinics (HVCs). Regular quality analysis is a major prerequisite of an HVC’s program, but few data have been reported so far. **Methods:** We retrospectively collected patients with isolated, native aortic valve stenosis who had been visited in our HVC at least once between 2021 and 2024. For each outpatient visit, symptoms, physical examination, echocardiographic data, complementary tests, and indications were acquired. Also, adverse events (hospitalization, unplanned procedures, and death) were retrieved. **Results:** A total of 320 patients were included. Mean visits/patient ratio was 1.2. At the first visit, 69.7% already had severe aortic stenosis, and severe symptoms (NYHA ≥ III) were evident in 24.4%. In addition, 26.5%, 59.1%, 12.8%, and 1.6% were in Généreux stage I, II, III, and IV, respectively. Overall, 197 (78.5%) and 54 (21.5%) patients received an indication for transcatheter AVR and surgical AVR, respectively. AVR-free survival was 46%, 23%, and 6% at 6, 12, and 24 months, respectively (mean 8.8 months CI95% 7.7–9.9). Adverse event-free survival was 97.2%, 95.5%, and 85% at 3, 6, and 12 months, respectively. **Conclusions:** Patients referred to our HVC already had an advanced disease with cardiac damage. Transcatheter AVR was mostly indicated, and it showed excellent short-term results. A low rate of adverse events was seen among patients in follow-up, but the odds of receiving AVR were high and driven by Généreux’s stage. Despite these favorable results, further efforts to sensitize earlier patient referral should be made.

## 1. Introduction

Aortic valve stenosis is the most common valvular heart disease (VHD), and it is a major public health burden [1,2]. Surgical and transcatheter aortic valve replacements are the only effective therapies [3,4].

According to current guidelines, management of VHD should be carried out in Heart Valve Centers with dedicated Heart Valve Clinics (HVCs) [3,4]. This recommendation arises from the growing complexity of both management and decisional processes, which in turn are result of the increasing prevalence of old, frail patients with multiple comorbidities. An accurate risk stratification, a detailed clinical framework, and tailored therapy are equally needed for these patients. Also, understanding the risk/benefit ratio as well as choosing the best treatment option (conventional surgery vs. minimally invasive surgery vs. transcatheter therapy) may be challenging in some cases [5].

The HVCs-provided standardized, multidisciplinary care sorts out these issues. It aims at simplifying the management of patients with VHD, supplying evidence-based care, and favoring timely access to consultations and diagnostic tests in order to improve quality of care [5,6].

However, in the real-world clinical practice of high-volume tertiary hospitals, outcomes may be jeopardized by several difficulties: satisfying the exploding burden of outpatient visits, avoiding unnecessary consultations, setting up an appropriate follow-up program, guaranteeing a fast delivery of second-level exams, and providing the best therapy for each patient. These are just a few of the problems that every HVC must deal with daily.

So, a systematic and regular quality analysis is a critical requisite of a valued HVC program. This study sought to provide real-world data of aortic stenosis patients’ flow, management, and outcomes followed in a high-volume HVC.

## 2. Materials and Methods

### 2.1. Heart Valve Clinic Patient Management and Flow

As a secondary-level outpatient clinic, the HVC is regulated by access criteria. Indeed, only patients with severe VHD (symptomatic or not) or moderate VHD with discordance between symptoms and echocardiographic findings are eligible for a medical visit [7]. Also, congenital heart valve disease, infective endocarditis, advanced heart failure, and life expectancy less than 1 year are exclusion criteria, as they are best managed in other dedicated programs.

Patients are referred to the HVC either by outpatient and inpatient care specialists filling out an electronic form in which the urgency can also be specified (low priority: to be visited within 3 months vs. high priority: within 3 weeks) [7].

During the first visit, a multidisciplinary team (cardiac surgeon, interventional cardiologist, and a cardiologist expert in cardiac imaging, with the support of a trained nurse) takes charge of each clinical case [7,8]. Past medical history, symptoms, physical examination, and pharmacological therapy are collected in an electronic database. A screening frailty evaluation is systematically performed. If the patient is considered at risk of frailty, a geriatrician proceeds with a detailed examination. At each visit, a 12-lead EKG and a comprehensive echocardiographic examination on the basis of a standardized examination protocol, including M-Mode, 2D echocardiography, conventional, and color Doppler, are performed. Cardiac damage is classified according to the stage system proposed by Gènèreux et al. [9]. In particular, patients were classified according to the criteria of the worst (i.e., highest) stage present. When necessary, complementary exams (i.e., exercise test/dobutamine echo-stress in asymptomatic patients or transesophageal echocardiogram) are carried out.

Patients who have an indication for treatment (surgical vs. percutaneous) undergo a specific preoperative work-up. This may include either a coronary angiography or an EKG-gated cardiac computed tomography for patients who need surgical aortic valve replacement (S-AVR) or a full-body computed tomography (including coronary assessment) for patients who are candidates for transcatheter aortic valve replacement (T-AVR).

Challenging cases may need a wider Heart Team discussion to decide on the best strategy. After the procedure, patients are followed up in dedicated outpatient clinics in order to not burden the HVC. Patients who have not yet met an indication for an invasive procedure are planned for further follow-up. Instead, patients who have an indication but are not suitable (i.e., prohibitive procedural risk) for any invasive treatment are managed with medical therapy.

The importance of early symptom onset referral, risk factor control, and appropriate oral hygiene are the focus of constant patient and family education.

Finally, patients who are not found to meet the HVC’s access criteria (i.e., those with an overestimated VHD) are back referred to their primary care physician.

### 2.2. Study Population and Data Acquisition

Patients with isolated, native aortic valve stenosis who were seen in our HVC between January 2021 and May 2024 were included in this study. Data were retrospectively recovered from the institutional electronic database. For each outpatient visit, symptoms, physical examination, echocardiographic data, complementary tests, and type of indication were acquired. Also, adverse events (hospitalization, unplanned procedures, death) were retrieved from outpatient visits’ reports, discharge letters, and phone interviews with either patients or referring physicians. Finally, 30-day outcomes of patients who underwent S-AVR or T-AVR were collected as well.

This study has been approved by the institutional review board (ID 7303). Due to its retrospective and observational design, informed consent was waived.

### 2.3. Statistical Analysis

Continuous variables are shown as mean ± standard deviation if normally distributed and as median (first–third quartile) otherwise. Percentages are used to describe categorical variables. The Kolmogorov–Smirnov test was used to check the normality/skewness of continuous variables before further analysis.

For comparing the means of two independent samples, Student’s t-test was used. The Chi-squared test was used to compare proportions between two independent samples.

Kaplan–Meier curves and log-rank tests were used to compare the overall event-free survival. Events were the occurrence of aortic valve replacement, either surgically or percutaneously, and the occurrence of one (or more) of the following: hospitalization, unplanned procedure, or death. Also, survival was analyzed according to Généreux’s stage. A Multivariable Cox proportional hazards model was used to determine the independent association between survival and variables chosen on the basis of their clinical relevance or statistical significance at univariate analysis. A *p*-value of 0.05 was considered to indicate statistical significance. Statistical analysis was performed with MedCalc vers. 22.0 (MedCalc Software Ltd., 8400 Ostend, Belgium).

## 3. Results

A total of 320 patients with isolated aortic valve stenosis underwent at least one visit during the study time frame and were included in this registry.

Of these, fifty-five (17.2%), twelve (3.7%), and two (0.6%) patients had a second, third, and fourth outpatient visit, respectively. Overall, the mean visits/patient ratio was 1.2, and the mean number of visits/year was 2.0 ± 0.3. Baseline patient characteristics are described in Table 1.

**Figure 1:**
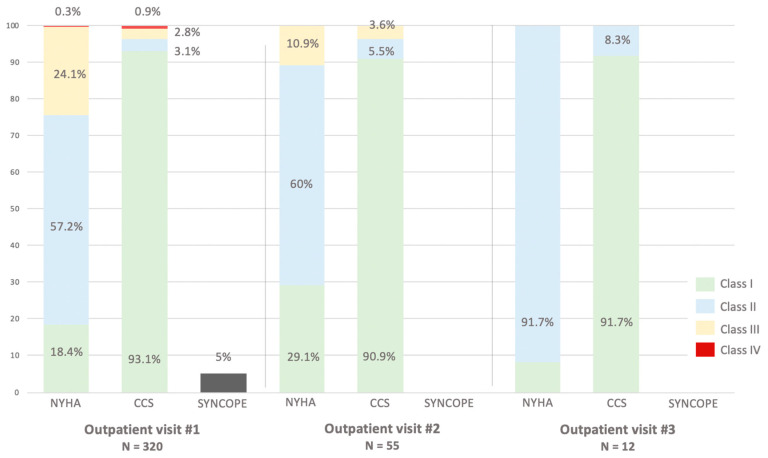
Symptoms distribution at different outpatient visits. Dyspnea and angina are described by the New York Heart Association (NYHA) and Canadian Cardiovascular Society (CCS) classifications, respectively.

At the time of the first visit, 69.7% of patients already had severe aortic valve stenosis (Table 2). Instead, aortic stenosis was found to be moderate to severe and moderate in 17.2% and 13.1% of patients, respectively. Severe symptoms (NYHA ≥ III) were evident in 24.4% of cases (Figure 1).

In addition, 26.5%, 59.1%, 12.8%, and 1.6% of patients were in Gènèreux stage I, II, III, and IV, respectively. No patient had Gènèreux grade 0 at the first visit. The main echocardiographic findings are reassumed in Table 2.

### 3.1. Indications

**Figure 2:**
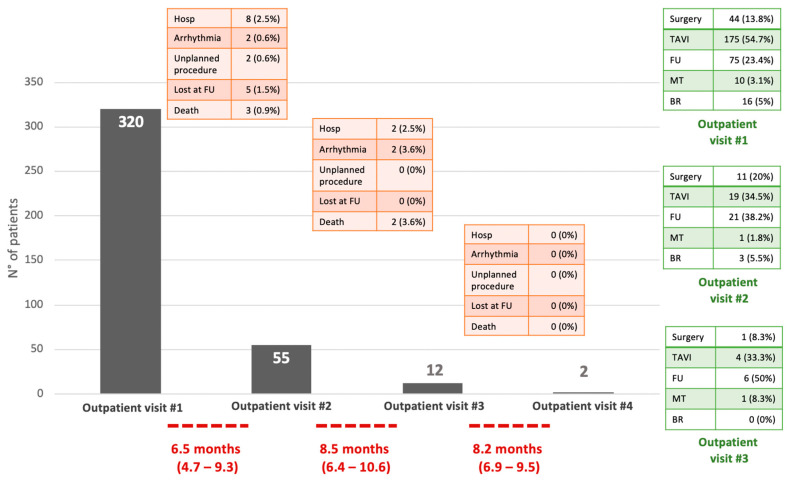
Flow, indications, and adverse events. In this modified histogram, each column corresponds to an outpatient visit. Green boxes (on the right) summarize indications. Red boxes describe adverse events and indicate patients lost to follow-up. The median time between visits is reported below in red. Although 75 and 21 patients received an indication for further follow-up at the 1st and 2nd visits, respectively, only 55 and 12 effectively received a follow-up visit. Of these 29 patients, 5 were lost to follow-up, 2 had an unplanned procedure, 9 received scheduled AVR due to the onset of symptoms as reported by the physician/satellite center, and 13 underwent medical therapy due to the identification of severe associated comorbidities. Hosp = hospitalization; FU = follow-up; MT = medical therapy; BR = back referral.

At the first visit, an indication for S-AVR or T-AVR was set for 44 (13.8%) and 175 (54.7%) patients, respectively. Seventy-five (23.4%) patients were scheduled for follow-up. Medical therapy was considered for 10 (3.1%) patients, and 16 (5%) were back-referred to their primary physician (Figure 2).

After a median of 6.5 months (4.7–9.3), 55 out of 75 patients had a follow-up visit. Of these, 19 (34.5%) received indication for T-AVR, 11 (20%) for S-AVR, 21 (38.2%) for further follow-up, 1 (1.8%) for medical therapy, and 3 (5.5%) for back-referral.

A third outpatient visit was necessary in 12 patients after a median of 8.5 months (6.4–10.6). Seven (58.3%) cases were managed conservatively with either an indication for further follow-up or medical therapy. In four (33.3%) and one (8.3%) patients T-AVR or S-AVR were indicated, respectively.

Finally, only two patients had a fourth visit and were scheduled either for T-AVR or follow-up.

### 3.2. Surgical and Transcatheter Aortic Valve Replacement Groups

In Table 3, a comparison of patients who received an indication for T-AVR vs. S-AVR is reported. T-AVR patients were significantly older (80.4 ± 6.3 vs. 69.3 ± 6.2 years, *p* < 0.01), had worse creatinine clearance (56.8 ± 20.8 vs. 83.1 ± 21.3 mL/min, *p* < 0.01), had a higher rate of previous cardiac surgery (8.6% vs. 0%, *p* = 0.02), and were more frequently frail (21.8% vs. 3.7%, *p* < 0.01). When considered together, patients who had received an indication for T-AVR or S-AVR had 22%, 62.5%, 12.7%, and 2.4% in Gènèreux stage I, II, III, and IV, respectively. An advanced Gènèreux grade (≥ III) was more common among T-AVR patients (36/197 vs. 2/54, *p* < 0.01).

**Figure 3:**
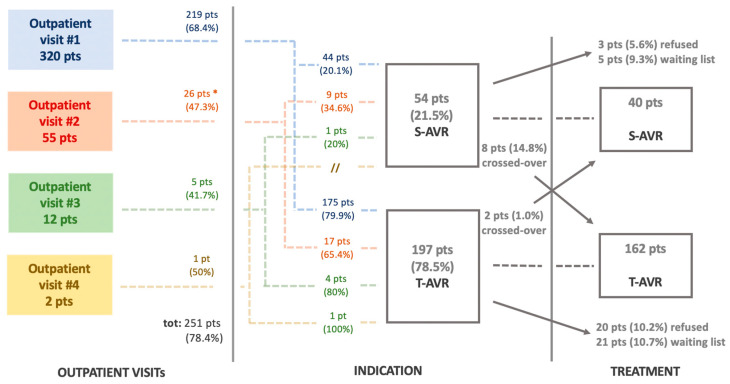
Flowchart showing procedural indication and treatment adherence. On the left, the number of patients for whom an invasive treatment was indicated is reported for each outpatient visit. In the middle, the cumulative number of patients who received an indication for surgical (S-AVR) or transcatheter (T-AVR) aortic valve replacement is shown. Finally, on the right, the number of patients who effectively received a treatment is reported. * Although 4 patients had received an indication for AVR at visit #1, they had a follow-up visit that confirmed the previous decision.

Of 54 patients for whom a surgical indication had been formulated, 40 (74.1%) actually received surgery after a median of 5.5 months (2.4–6.5). Indeed, three (5.6%) refused surgery, five (9.3%) were still on the waiting list, and eight (14.8%) had crossed over to T-AVR (Figure 3).

Of 197 patients who received an indication for transcatheter AVR, 162 (82.2%) had T-AVR after a median of 3.4 months (2.1–5.1). Twenty patients (10.2%) refused, twenty-one (10.7%) were still on waiting list, and two (1%) crossed over to surgery.

Overall, 30-day mortality was 2.5% (5/202). All these patients had received T-AVR.

### 3.3. Adverse Events and Survival Analysis

Further, five patients (1.6%) died while on the waiting list (two), in medical therapy (two), or during follow-up (one) after a median of 8.6 months (5.7–20.2) after the first outpatient visit. With the exception of one patient who died from septic shock during follow-up, all others could be considered cardiovascular deaths.

Ten patients (3.1%) had a hospitalization (five while on the waiting list and five during follow-up). Of these patients, two (0.6%) required an unplanned procedure. Finally, five patients (1.6%) were lost to follow-up.

**Figure 4:**
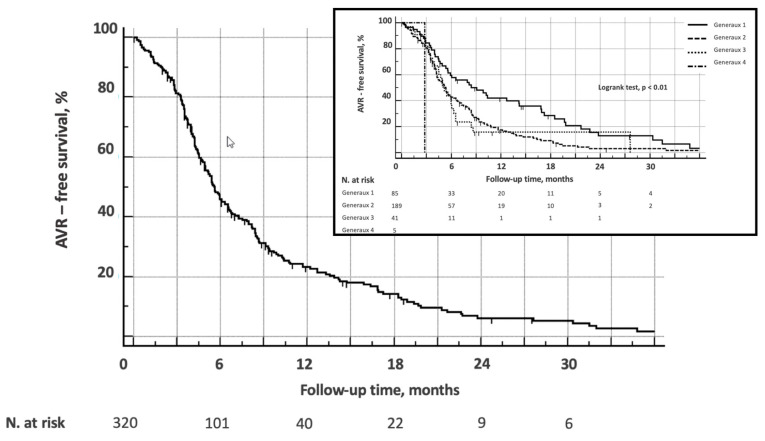
Kaplan–Meier analysis of surgical or transcatheter aortic valve replacement (AVR)—free survival for the entire study population. In the upper right-sided box, AVR-free survival is stratified according to the Généreux’s stage.

As shown in Figure 4, AVR-free survival was 46%, 23%, and 6% at 6, 12, and 24 months, respectively (mean 8.8 months, CI95% 7.7–9.9). When stratified by Gènèreux’s stage, patients with advanced grade had worse AVR-free survival (log-rank test, *p* < 0.01).

Cox proportional-hazards regression showed that age (for 1-year increase: 1.04, CI95% 1.02–1.06, *p* < 0.01), Gènèreux’s stage (1.33, CI95% 1.04–1.70, *p* = 0.03), and aortic stenosis severity (2.70, CI95% 1.92–3.80, *p* < 0.01) were significantly associated with an increased risk of T-AVR or S-AVR.

**Figure 5:**
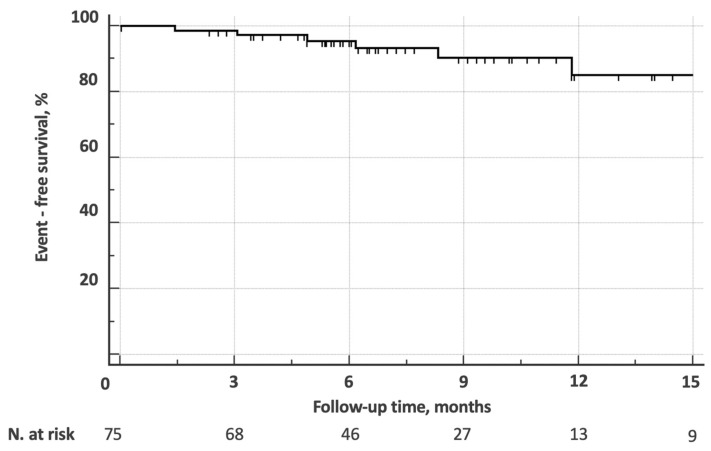
Kaplan–Meier analysis of adverse event (death, unplanned hospitalization, or procedure)—free survival for patients in follow-up.

Event-free (death, hospitalization, or unplanned procedure) survival for patients in follow-up was 97.2%, 95.5%, and 85% at 3, 6, and 12 months, respectively (Figure 5). Although a trend was perceivable, no predictive factor could be identified.

When considering only patients for whom follow-up was indicated after the first visit, AVR-free survival was 93.6%, 80.6%, and 22.9% at 6, 12, and 24 months, respectively, with no correlation with Gènèreux’s stage.

## 4. Discussion

Healthcare professionals specialized in the diagnosis, management, and treatment of patients with VHD are the core of the Heart Valve Clinic [6]. The aging population, the increasing VHD incidence, and the evidence that some patients did not receive guidelines-directed care led to the development of this management model [5,10,11]. The multidisciplinary, standardized, and guidelines-based approach of the HVC has several purposes [6,12]: (1) coordinate the practice of different specialists involved in the management of VHD patients, (2) prescribe and interpret diagnostic exams, (3) improve management and outcomes, simplifying access to invasive procedures, and (4) promote education with regard to medical therapy compliance and prompt symptoms onset reporting. Not least, periodic analysis of outcomes should be performed with the goal of achieving always better results. Indeed, slipping on the high number of patients referred to or on prolonged waits for either medical examinations or invasive treatments is concrete and may turn this virtuous tool into a boomerang.

Despite these observations, few heterogeneous real-world data regarding HVCs’ outcomes have been reported so far [5,11,13].

So, in this study we aimed at describing contemporary 3-year results of our HVC in the management of patients with native aortic valve stenosis.

Frequently, patients referred to our outpatient clinic were old-aged, with multiple cardiovascular risk factors and associated diseases. Also, one patient out of five was found to be clinically frail.

At the time of the first visit, 70% had severe aortic stenosis, and 25% complained of advanced symptoms. More importantly, patients presented some grade of cardiac damage, as highlighted by the Généreux stage. Not surprisingly, most patients received an indication for invasive treatment already at the first visit.

The number of patients who either did not have criteria for being managed by the HVC or who were not found to be eligible for either surgical or transcatheter AVR was limited. This result could be explained by the access criteria redefinition that we had performed to both avoid excessive HVC load and improve its appropriateness [7]. Also, the close collaboration with the network of referring centers played an essential educational role as it allowed avoiding unnecessary outpatient services, reducing both costs and visits waiting time.

One patient died while on follow-up, but he had moderate aortic stenosis, and death was due to septic shock. Five patients had an unplanned hospitalization, but only three for cardiovascular complications. None of them needed an unplanned procedure.

Follow-up was carried out on time, with no delays in the delivery of outpatient visits. As pointed out by Lancellotti and colleagues, the HVC workload depends on both the availability of personnel and facilities [6]. After an initial testing phase, the HVC was set once a week with seven visits of 45 min each. This model was found to be the most commonly used in the UK [14]. However, it could not be considered unchangeable. Indeed, it may always happen that a bigger number of visits are needed sometimes. So, the model should be flexible with more slots to be opened when necessary. Similarly, the duration of each visit is of non-negligible importance, and it should be preserved as it gives the possibility to provide adequate patient and family education.

Most patients received an indication for T-AVR. This was attributable to the baseline characteristics of patients referred to the HVC. Patients who received T-AVR were more clinically complex and presented with advanced cardiac damage and symptoms. This latter evidence could explain why S-AVR patients had to wait longer for the procedure to be performed.

It has been reported that an increase in Généreux’s stage is associated with higher 1-year mortality after transcatheter or surgical AVR, regardless of symptoms or ejection fraction [9]. So, patients in Généreux’s stages I, II, III, and IV have 9.2%, 14.4%, 21.3%, and 24.5% 1-year mortality, respectively [9]. Since timely referral to treatment is paramount [10], this evidence should not be ignored. In our experience we have found that for each increase in Généreux grade, the probability of treatment increased by 33%.

Other reports found that Généreux’s stage could predict the risk of hospitalization and its duration in the 6 months preceding T-AVR [15]. Although a trend was conceivable, the small number of events among patients in follow-up or waiting for a procedure did not make it possible to find an association between the Généreux’s stage and adverse outcomes.

In a seminal paper, Tastet et al. [16] found that cardiovascular mortality significantly rose when Généreux’s stage increased from I (25%) to II (44%). Accordingly, authors suggested considering patients for treatment before stage II was reached.

Similar results were described by Vollema and coauthors [17] in a population of patients with symptomatic aortic stenosis. They found that Généreux’s stage was associated with overall mortality and with the composite end point of death, stroke, and hospitalization.

In our registry, by the time of the first visit, patients already had cardiac damage. Further, most patients who received an indication to treatment were already in stage II or more. These results may be explained by the fact that the Généreux’s stage has not yet been absorbed by clinical practice, and referral still occurs too late. So, sharing these considerations with the network of referring hospitals and specialists, in order to send patients before advanced cardiac damage takes place, is of paramount importance.

Beyond the Généreux’s stage, HVC management itself improves patients’ prognosis. In a recent propensity-matched study on 2129 patients with moderate or severe asymptomatic aortic stenosis, Paolisso et al. found that those who were followed by an HVC had reduced both all-cause mortality and cardiovascular death compared to standard care [18]. The higher visit/year ratio (1.6 vs. 0.8), longer duration of each visit (40 vs. 20 min), and higher number of both instrumental and laboratory examinations were advocated to explain those excellent results, which distinguish the good clinical practice of an HVC. Longer visit durations give the opportunity to fully inform the patient and his/her family of the disease, to educate in timely symptoms reporting, and to anticipate the possibility of an invasive treatment, illustrating the pros and cons of surgical vs. percutaneous AVR.

The higher number of visits per year derives from the evidence of an advanced aortic stenosis and reflects the intention to early identify symptoms or cardiac damage worsening in order to perform AVR with no delay. Rudolph and colleagues [19] have recently compared 594 patients with severe aortic stenosis followed by an HVC with 196 patients treated in satellite centers. Although HVC patients showed higher clinical complexity, they underwent AVR more frequently (52.6% vs. 31.3% after 3 months), and 1-year survival was higher (90.5% vs. 84.8%; *p* = 0.047). These outcomes reflect a substantially greater aggressiveness in the HVC management of VHDs, even in challenging patients.

## 5. Conclusions

In this single-center study we reported the real-world outcomes of patients with aortic valve stenosis followed in our HVC during a 3-year time frame. We found that most referred patients were complex, and, uppermost, they already had cardiac damage at the time of the first visit. Transcatheter AVR was indicated in the majority of cases with excellent short-term results. Patients in follow-up experienced a low rate of adverse events, but the probability to undergo treatment was high and associated with Généreux’s stage. Despite these results, a further effort to sensitize specialists and satellite centers for earlier patient referral should be made.

## Figures and Tables

**Table 1:** Baseline characteristics of the study population.

Variable	Study Population (*n* = 320)
Age, years	77.5 ± 8.5
Women	177 (55)
BMI, kg/m^2^	27.1 ± 4.3
BSA, m^2^	1.8 ± 0.2
Diabetes	82 (25.6)
Hypertension	270 (84.4)
Active Smoker	44 (13.8)
Dyslipidemia	210 (65.6)
COPD	63 (19.7)
PAD	27 (8.4)
Dialysis	11 (3.4)
Malignancy *	10 (3.1)
Previous PCI	64 (20)
Previous PMK	23 (7.2)
Previous cardiac surgery	22 (6.9)
CABG	19 (5.9)
MVR	3 (0.9)
Frailty	
Fit	164 (51.3)
Pre-Frail	96 (30)
Frail	60 (18.8)
EuroSCORE II	2.0 (1.3–3.2)

Continuous variables are reported as mean ± standard deviation or median (first–third interquartile), as appropriate. Categorical variables are reported as number (%). BMI = body mass index; BSA = body surface area; CABG = Coronary Artery Bypass Graft; COPD = Chronic Obstructive Pulmonary Disease; MVR = Mitral Valve Replacement; PAD = Peripheral Artery Disease; PCI = Percutaneous Coronary Intervention; PMK = Pacemaker; * Only active malignancies with expected survival > 12 months are included.

**Table 2:** Echocardiographic findings.

Variable	Study Population (N = 320)
Aortic Stenosis	
Moderate	42 (13.1)
Moderate to severe	55 (17.2)
Severe	223 (69.7)
AVA, cm^2^	0.9 ± 0.2
AV max PG, mmHg	68 (53–84)
AV mean PG, mmHg	44 (32–54)
AV peak velocity, m/s	4.1 ± 0.8
VTI ratio	0.2 ± 0.1
EF, %	60.0 ± 9.6
LV septum, mm	13.3 ± 2.2
LV posterior wall, mm	11.3 ± 2.6
LV end-diastolic volume, mL	91.7 ± 32.1
LV end-systolic volume, mL	38.2 ± 20.8
PAPs	38.2 ± 11.5
RV dysfunction	21 (6)
TAPSE	21.1 ± 3.7
Gènèreux’s stage	
I	85 (26.5)
II	189 (59.1)
III	41 (12.8)
IV	5 (1.6)

Continuous variables are reported as mean ± standard deviation or median (first–third interquartile), as appropriate. Categorical variables are reported as number (%). AVA = Aortic valve area; AV = aortic valve; PG = Pressure Gradient; VTI = Velocity Time Integral; EF = ejection fraction; LV = Left Ventricle; PAPS = Systolic Pulmonary Artery Pressure; RV = Right Ventricle; TAPSE = Tricuspid Annular Plane Systolic Excursion.

**Table 3:** Clinical and echocardiographic comparison of patients who received an indication for surgical (S-AVR) versus transcatheter (T-AVR) aortic valve replacement.

Variable	S-AVR (N = 54)	T-AVR (N = 197)	*p* Value
Age, years	69.3 ± 6.2	80.4 ± 6.3	<0.01
Women	23 (42.6)	116 (58.9)	0.04
BMI, kg/m^2^	27.5 ± 4.2	27.1 ± 4.4	0.55
BSA, m^2^	1.9 ± 0.2	1.8 ± 0.2	<0.01
Diabetes	13 (24.1)	53 (26.9)	0.73
Hypertension	48 (88.9)	165 (83.8)	0.40
Active Smoker	8 (14.8)	19 (9.6)	0.32
Dyslipidemia	35 (64.8)	129 (65.5)	1
COPD	6 (11.1)	39 (19.8)	0.14
PAD	3 (5.6)	14 (7.1)	0.69
Creatinine Clearance, mL/min	83.1 ± 21.3	56.8 ± 20.8	<0.01
Dialysis	2 (3.7)	6 (3)	0.81
Malignancy *	0 (0)	8 (4.1)	0.29
Previous PCI	7 (13)	40 (20.3)	0.24
Previous PMK	0 (0)	16 (8.1)	0.03
Previous cardiac surgery	0 (0)	17 (8.6)	0.02
Frailty			<0.01
Fit	45 (83.3)	80 (40.6)	
Pre-Frail	7 (13)	74 (37.6)	
Frail	2 (3.7)	43 (21.8)	
EuroSCORE II	1.3 (1.0–1.9)	2.2 (1.5–3.9)	
Aortic Stenosis: Grade			
Moderate	1 (1.9)	0 (0)	0.21
Moderate to Severe	6 (11.1)	15 (7.6)	0.41
Severe	46 (85.2)	181 (91.9)	0.19
AVA, cm^2^	0.8 ± 0.2	0.8 ± 0.2	1
AV mean PG, mmHg	51.7 ± 15.1	47.5 ± 14.2	0.06
AV peak velocity, m/s	4.4 ± 0.7	4.2 ± 0.7	0.06
EF, %	61.4 ± 8.5	60.1 ± 9.8	0.38
PAPs	35.4 ± 8.8	39.6 ± 12.5	0.02
Gènèreux’s stage			
I	13 (24.1)	43 (21.8)	0.72
II	39 (72.2)	118 (59.9)	0.11
III	2 (3.7)	30 (15.2)	0.02
IV	0 (0)	6 (3)	0.35

Continuous variables are reported as mean ± standard deviation or median (first–third interquartile), as appropriate. Categorical variables are reported as number (%). AVA = Aortic valve area; AV = aortic valve; EF = ejection fraction; PAPs = Systolic Pulmonary Artery Pressure; BMI = body mass index; BSA = body surface area; COPD = Chronic Obstructive Pulmonary Disease; PAD = Peripheral Artery Disease; PCI = Percutaneous Coronary Intervention; PMK = Pacemaker; * Only active malignancies with prognosis > 12 months are included.

## Data Availability

The original contributions presented in this study are included in the article. Further inquiries can be directed to the corresponding author(s).
